# The UCSC repeat browser allows discovery and visualization of evolutionary conflict across repeat families

**DOI:** 10.1186/s13100-020-00208-w

**Published:** 2020-03-31

**Authors:** Jason D. Fernandes, Armando Zamudio-Hurtado, Hiram Clawson, W. James Kent, David Haussler, Sofie R. Salama, Maximilian Haeussler

**Affiliations:** 1 Genomics Institute, University of California, Santa Cruz, USA; 2 Department of Biomolecular Engineering, University of California, Santa Cruz, USA; 3 Howard Hughes Medical Institute, University of California, Santa Cruz, USA; 4 Big Data to Knowledge Program, California State University, Monterey Bay, USA

**Keywords:** Repeats, Retrotransposon, Genomics, Krab zinc finger proteins, Evolution

## Abstract

**Background:**

Nearly half the human genome consists of repeat elements, most of which are retrotransposons, and many of which play important biological roles. However repeat elements pose several unique challenges to current bioinformatic analyses and visualization tools, as short repeat sequences can map to multiple genomic loci resulting in their misclassification and misinterpretation. In fact, sequence data mapping to repeat elements are often discarded from analysis pipelines. Therefore, there is a continued need for standardized tools and techniques to interpret genomic data of repeats.

**Results:**

We present the UCSC Repeat Browser, which consists of a complete set of human repeat reference sequences derived from annotations made by the commonly used program RepeatMasker. The UCSC Repeat Browser also provides an alignment from the human genome to these references, uses it to map the standard human genome annotation tracks, and presents all of them as a comprehensive interface to facilitate work with repetitive elements. It also provides processed tracks of multiple publicly available datasets of particular interest to the repeat community, including ChIP-seq datasets for KRAB Zinc Finger Proteins (KZNFs) – a family of proteins known to bind and repress certain classes of repeats. We used the UCSC Repeat Browser in combination with these datasets, as well as RepeatMasker annotations in several non-human primates, to trace the independent trajectories of species-specific evolutionary battles between LINE 1 retroelements and their repressors. Furthermore, we document at https://repeatbrowser.ucsc.edu how researchers can map their own human genome annotations to these reference repeat sequences.

**Conclusions:**

The UCSC Repeat Browser allows easy and intuitive visualization of genomic data on consensus repeat elements, circumventing the problem of multi-mapping, in which sequencing reads of repeat elements map to multiple locations on the human genome. By developing a reference consensus, multiple datasets and annotation tracks can easily be overlaid to reveal complex evolutionary histories of repeats in a single interactive window. Specifically, we use this approach to retrace the history of several primate specific LINE-1 families across apes, and discover several species-specific routes of evolution that correlate with the emergence and binding of KZNFs.

## Introduction

Transposable elements are significant drivers of eukaryotic genome evolution. In humans and other primates, transposons constitute nearly half the genome; the majority of these repeat elements are retrotransposons, although some DNA transposons are also present. Despite the high repeat content of the human genome, many genomic analyses struggle to deal with these regions as sequencing reads can often be assigned nearly equally well to multiple regions in the genome. Masking or filtering these reads is often considered a “conservative” approach in that it avoids mis-assigning the genomic location of a read, but it prevents the discovery of potentially important biological functions of repeat elements [1]. Indeed, many repeats already have established roles in important biological processes, complex behavioral phenotypes, and disease [2–5].

**Fig. 1:**
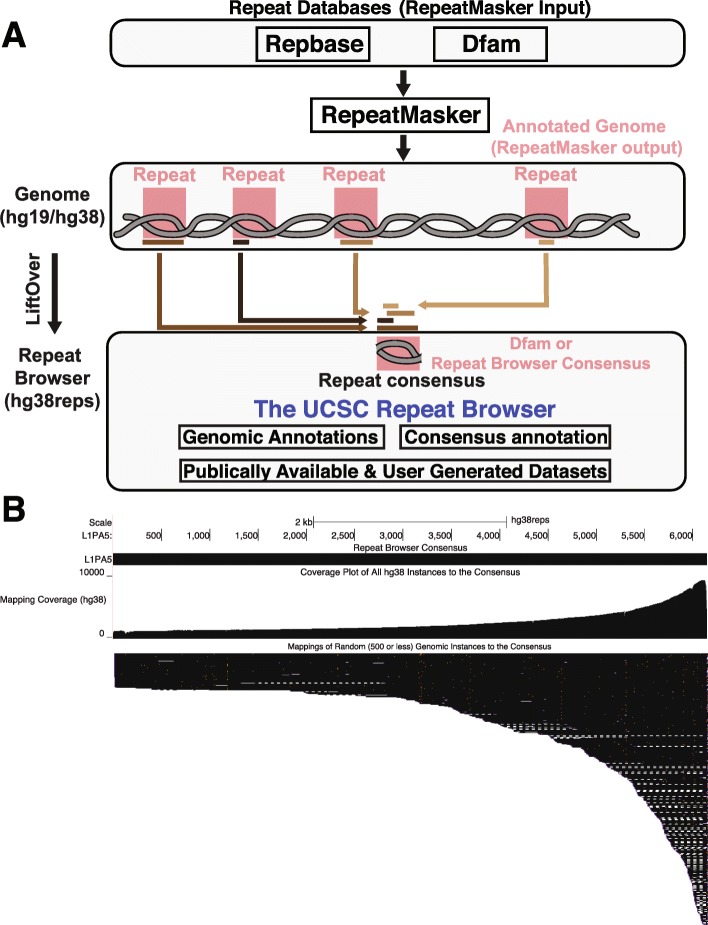
Building the UCSC Repeat Browser. **a** Workflow for building the UCSC Repeat Browser. Repeats are annotated on the human genome by the program RepeatMasker using a database of input sequences and repeat models from RepBase and Dfam. These genomic annotations are then mapped to representative consensuses of every repeat family (consensuses are taken directly from Dfam or built from RepeatMasker output). Genomic data (brown bars all shades) such as gene annotations or user-generated data can be “lifted” from the human genome to the Repeat Browser. **b** Mapping of individual L1PA5 instances to the consensus. A majority of L1PA5 sequences in the human genome only contain the 3′ end as evidenced by the coverage per base (mapping coverage) and alignments of individual instances (mapping alignments of 500 randomly selected elements)

One of the major challenges in proper repeat-analysis is establishing a set of standardized sequences, nomenclature and annotation sets that can be universally understood by the scientific community. The most commonly used databases and tools to study repeats are Repbase [6] and RepeatMasker [7]. Repbase began as a hand-curated list in 1992 of 53 prototypic repeat sequences identified in the human genome [8]. By 2015, it contained more than 38,000 sequences in 134 species [6], making curation and comprehension of each repeat family a daunting challenge. RepeatMasker is a program that screens DNA (e.g. a newly sequenced genome) for repeat elements by filtering, merging and joining human genome alignment matches based on a database of partial repeat sequences. The strategies are optimized for the different classes of repeats. For instance, some full length repeat elements such as LINE-1 elements are built after alignments for sequences of smaller subparts (e.g. 5′ and 3′ UTRs) are identified (Fig. 1a).

One option for the sequence database as the basis of these alignments is RepBase (RepBase RepeatMasker Edition). More recent versions of RepeatMasker utilize the Dfam database [9], an open database of hidden Markov model profiles of repeat elements, as RepBase now requires a commercial license. All sequences and RepeatMasker annotations described in the following are therefore based on Dfam.

Although a variety of tools and methods already exist to study repeats [10], tools to dynamically visualize genomic data utilizing existing annotation sets on repeats (e.g. protein coding regions, conservation with other sequences and the list of matches in the genome) are still needed. Generating and mapping to a consensus version of individual repeats has proven successful in illustrating novel biological features of transposon insertions, but has largely been limited to static visualizations on targeted elements of interest and specific families of these repeats [11–13].

Here we present the UCSC Repeat Browser, which simplifies analysis of genomic data on repeats by using consensus sequences for all human repeat element classifications within RepeatMasker. The Repeat Browser allows “lifting” of human genomic data to these consensuses where they can be analyzed in conjunction with a precomputed set of comprehensive annotations in an interactive genomic browser environment (Fig. 1). Further, we demonstrate the utility of the Repeat Browser in illustrating how a primate specific class of retrotransposons has evolved to delete portions of their own sequence that likely allow them to evade the binding of repressors.

## Implementation

### Generating reference sequences for human repeats

We ran RepeatMasker using Dfam on the two most recent assemblies of the human genome (hg19 and hg38) and generated a list of repeat families (excluding the simple repeats) annotated on the genome. For families that had a Dfam equivalent (Table S1), we utilized the Dfam sequence as a consensus. For families that have no exact Dfam equivalent (Table S1), where the repeats are annotated by joining alignments of partial sequences, we generated our own consensuses.

To do so, we downloaded all nucleotide sequences and their annotations in the RepeatMasker annotation track on the UCSC Human Genome Browser (hg38). We observed that extremely long repeats tended to represent recombination or misannotation events and therefore removed the longest 1% of sequences in all classes. We then aligned the 50 longest remaining sequences of each class, as this produced a tractable number of sequences that allowed manual inspection of each alignment, and because insertions relative to the consensus are difficult to show on shorter sequences. For each repeat family, these fifty sequences were realigned with MUSCLE [14] to create a consensus sequence. Each of these consensus sequences was then stored as a “reference” in the Repeat Browser in a manner analogous to a single chromosome on the UCSC Human Genome Browser [15, 16]. Each alignment is provided as a link in a “consensus alignment” track for additional visual inspection by the user.

In addition, we added two consensus sequences manually, HERVH-full and HERVK-full, which represent full length reconstructions (internal regions + LTR) that have been shown to produce viral particles [17, 18]. We added these sequences as HERV-K and HERV-H are well studied ERVs with established biological roles, and users may wish to analyze these sequences in the context of a full ERV instead of the smaller subparts (ERV internal regions and LTRs) that RepeatMasker does not join in its final output. However the Repeat Browser also contains the Dfam consensuses for these elements (HERVH and HERVK, which correspond only to the internal regions of the ERVs as well as their corresponding LTRs) meaning that data mapped to any of these regions in the human genome corresponds to multiple consensus sequences in the Repeat Browser.

We also generated a track with existing Dfam annotations for the Dfam consensus sequences. For our custom generated consensuses, we aligned the RepeatMasker Peptide Library [19] (filtered only to include peptides derived from human repeats) with tBLASTn [20] and took the highest scoring hits to automatically annotate ORFs. We also ran Tandem Repeats Finder [21] on these consensus sequences to annotate tandem repeats on each consensus (e.g. variable nucleotide tandem repeat regions within repeats). The combination of these consensus sequences (Dfam and custom Repeat Browser sequences) serve as the “genome” for all repeat content and are collectively called “hg38reps”. The script to build these consensus sequences is available as part of the “buildSeqs” step in the Repeat Browser source code.

### Building LiftOver chains between the genome and repeat browser

In order to map a coordinate set on the human genome to coordinates on our consensus sequences on the Repeat Browser (a process more generally known as “lifting” [22]), we first mapped every consensus to all annotated genomic instances (both hg19 and hg38) of the same type using BLAT [23] (Fig. 1a). From this process, we generated a coverage plot illustrating where the instances align to the consensus (Fig. 1b). For example, the primate-specific LINE-1 sub-family, L1PA5, shows the distribution expected for recently active LINE elements: most individual L1PA5 instances, are defective short 3′ truncations, with only a few near full length elements containing the 5′ portion. Therefore the 3′ end of the consensus is found relatively often across the human genome (Fig. 1b). Using these alignments between consensus and genome, we then generated liftOver chains that allow genomic data to be visualized on the Repeat Browser. The script to align the consensuses and build the liftOver chains files is provided as the “buildLiftOver” step in the Repeat Browser source code.

### Lifting of genomic annotations

**Fig. 2:**
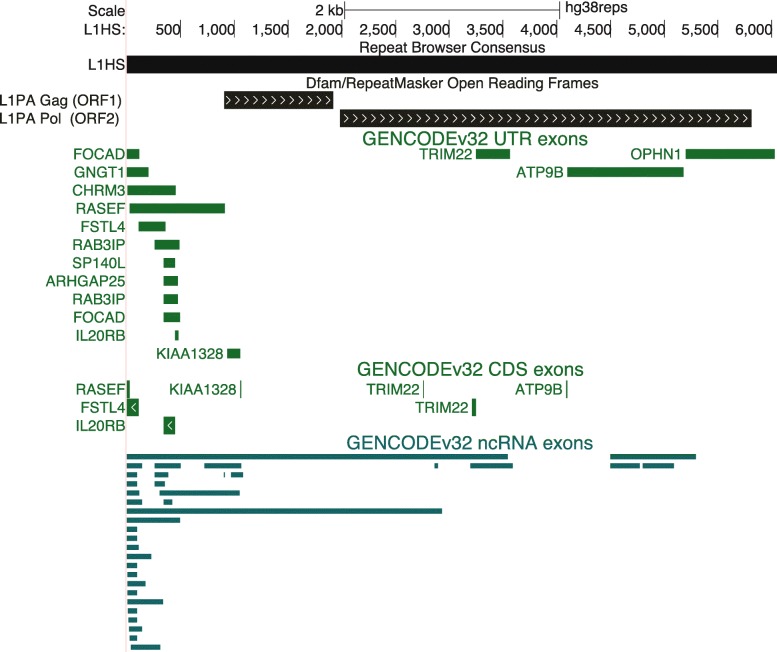
Mapping of existing annotations and detection of repeat features. Annotation sets (e.g. GENCODEv32) that intersect RepeatMasker annotations were lifted from hg38 to the Repeat Browser consensuses. Exons were categorized as coding, UTRs of protein coding genes, and exons of non-coding genes. Shown here are all genes that contain L1HS sequence as well as annotated ORFs (detected by tblastn)

These liftOver chains allow us to map existing genome annotation sets from hg19 and hg38 to the Repeat Browser consensus sequences. We lifted several standard genomic annotations to the Repeat Browser (Table 1); for instance, lifting of human gene annotation sets (e.g. GENCODE [24]) allows visualization of genes which contain repeat sequence within them. For example, L1HS sequences (Fig. 2) that have been incorporated into protein coding genes tend to derive from the untranslated regions (UTRs) of the repeats, and also tend to incorporate into the UTRs of the protein coding genes. Conversely, ncRNAs tend to contain the 5’UTR of L1HS elements and can span longer portions of the consensus. The result of these procedures produces a fully annotated and interactive consensus sequence that requires minimal prior knowledge of the genomic organization of the repeat being analyzed and at the same time allows lifting of any genome annotation from either hg19 or hg38 available at online at repeatbrowser.ucsc.edu.

**Table 1:** List of Tracks available on the Repeat Browser

Track	Description
Mapping Alignments	Alignments of each individual repeat instance in hg38 back to the Repeat Browser consensus.
Mapping Coverage	A coverage plot for the mapping alignments from the above track.
Annotations (ORFs and UTRs)	Gene annotations of the repeat element as annotated in Dfam or detected by searching the RepeatMasker peptide library.
Self Alignments	Alignment of all other Repeat Browser Consensuses against the currently viewed consensus.
GENCODEv32	Alignments of GENCODEv32 annotated coding sequences, UTRs of protein-coding genes, and ncRNAs to the RepeatBrowser.
Tandem Repeats	Detected tandem sequence repeats within the consensus full-length repeat elements.
ENCODE Tracks	DNAse hypersensitive sites, histone marks (UW collection) and TF ChIP-seq (TFBS collection) from ENCODE lifted to the Repeat Browser.
KZNF Tracks (Imbeault/Trono 2017 & Schmittges/Hughes 2016)	Lifting of reprocessed data from large KZNF ChIP-seq screens.
TF Differentiation Data (Tsankov 2014)	Lifting of large scale ChIP-seq dataset of TFs involved in differentiation of iPSCs to multiple cell types.
Stem Cell Naive State Data (Theunissen 2016)	Lifting of H3K9me3 and Kap1 ChIP-SEQ from primed and naïve human pluripotent stem cells.

### Mapping of existing genomic datasets

**Fig. 3:**
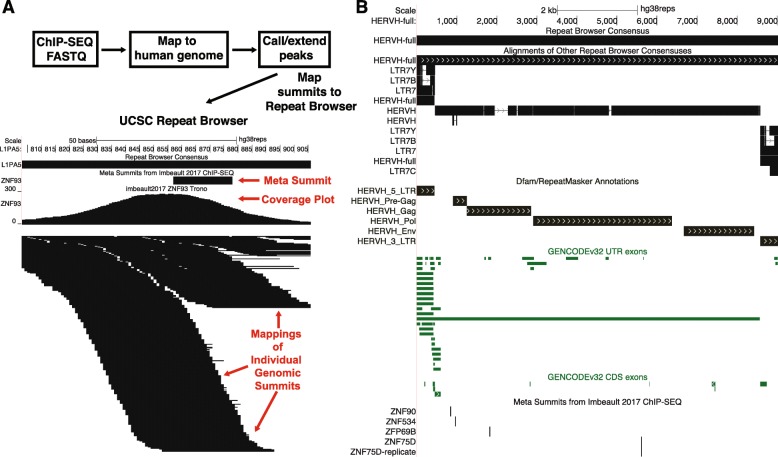
Mapping of KZNF ChIP-seq data to the UCSC Repeat Browser. **a** Workflow for analyzing KZNF ChIP-seq. Data from existing collections was downloaded from SRA, analyzed via standard ChIP-seq workflows and the resulting summits mapped back to the RB for analysis. Mapping of individual summits produces a “meta-summit” (red arrow) that can be used for downstream analysis and which is stored separately in another annotation track. **b** Example of a repeat family, HERVH-full (a reconstituted primate endogenous retrovirus containing both LTRs and the internal region) with lifted annotations and datasets. Shown are aligments to other Repeat Browser Consensuses (e.g. solo LTRs), tracks of repeat annotations, gene overlaps, and KZNF meta-summits

We also mapped genomic loci bound by histone-modifying enzymes from ENCODE datasets [25] as well as large-scale ChIP-seq collections KRAB Zinc Finger Proteins (KZNFs) [26, 27] to the Repeat Browser. KZNFs are particularly compelling factors as they engage in evolutionary “arms races” in which KZNFs evolve unique DNA binding properties to bind and repress retrotransposons [11, 28]. These retrotransposons then accumulate mutations that allow evasion of KZNF-mediated repression [11]. In order to map this ChIP-seq data to the Repeat Browser, we first downloaded raw ChIP-seq reads from the Sequence Read Archive (SRA) [29], mapped them to the reference genome (hg19) using bowtie2 [30] and called peaks using macs2 [31] (Fig. 3a). After this standard genomic mapping and peak calling, we then took the summits of these peaks, extended them by 5 nt in both directions, and lifted them to the Repeat Browser consensus sequences. In essence, this approach leverages each repeat instance as a technical replicate, with the mapping to the consensus representing a combination of many genomic “replicates” (Fig. 3a) of DNA binding summits called on individual instances of a repeat family that individually produce a noisy set of mappings; however hundreds of them combined yield a clear overall signal, better identifying the actual binding site. We call this “summit of summits” (obtained by combining the summits on individual transposon instances into a single summit on the Repeat Browser consensus) the “meta-summit”. In order to determine these “meta-summits”, we used a peak caller (macs2) on the repeat consensus to generate a list of “meta-summits” which represent the most likely location of the DNA binding site for a specific DNA-binding factor. We then generated a track which summarizes these meta-summits for each consensus sequence allowing easy and quick determination of factors with correlated binding patterns (Fig. 3b; visualized on a full length sequence of Human Endogenous Retrovirus H, HERV-H). These meta-summits serve as a quick summary of which factors bind which consensus sequence. These meta-summits can then be investigated more deeply by examining the coverage and mappings of each individual factor which are provided as separate tracks.

## Results

### Comparative analysis of L1PA elements

**Fig. 4:**
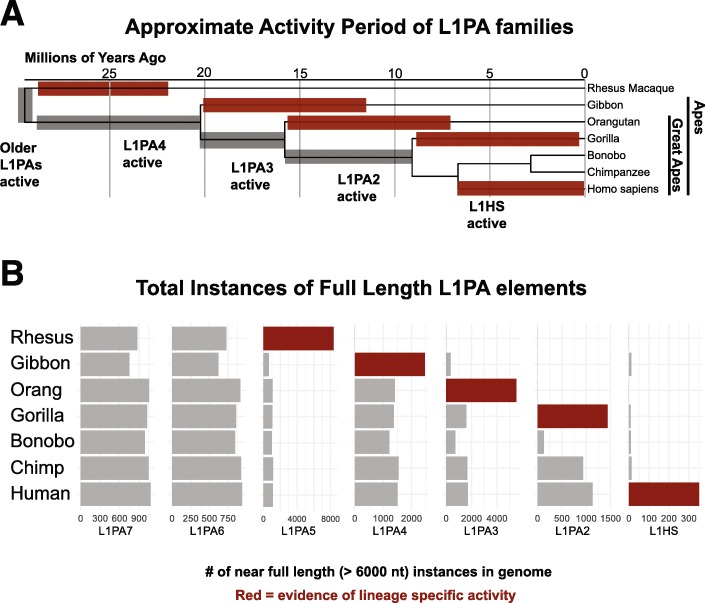
Comparative analysis of L1PA elements. **a** Phylogeny and nomenclature of L1PA elements. Older elements have higher numbers and families can expand in a manner that will be conserved between species (grey) or lineage-specific (red). **b** Counts of near full length L1PA instances (> 6000 nt) extracted from UCSC Repeat Masker tracks. Note for Rhesus (rheMac10), L1PA5 counts represent a sum of rhesus-specific elements (labeled as L1PA5 in RepBase, L1_RS* by RepeatMasker). Families in red expand greatly compared to families in grey, providing evidence of lineage-specific expansion

In order to demonstrate the power of the UCSC Repeat Browser, we studied the evolution of recent L1PA families. The L1PA lineage is a group of LINE-1 retrotransposon families specific to primates. These elements are fully autonomous, encoding proteins ORF1 (Gag in the RepeatMasker Peptide Library) and ORF2 (Pol in the RepeatMasker Peptide Library) responsible for reverse transcription and re-integration of the retrotransposon. L1PA families evolve in bursts; higher numbers (e.g. L1PA17) indicate ancient evolutionary origins, as evidenced by shared copies across species (Fig. 4a). Lower numbers indicate more recent activity and are derived from the older, higher number families (note L1PA1 is also known as L1HS, human-specific) [32]. Although this nomenclature generally corresponds to speciation events on the phylogenetic tree of the hosts of L1PA retrotransposons, many families have had overlapping periods of activity meaning that the correspondence is not exact [33].

### Comparison of primate repeat elements reveals a large number of gibbon specific L1PA4 elements

In order to trace the evolution of L1PAs in different species, we downloaded the complete sequences for every L1PA7 and younger L1PA family, as annotated in their UCSC Genome Browser RepeatMasker tracks, in rhesus macaque (rheMac10), gibbon (nomLeu3), orangutan (ponAbe3), chimp (panTro6), gorilla (gorGor5), bonobo (panPan2) and human (hg38). We further restricted our analysis to near full-length elements by filtering out elements less than 6000 nucleotides in length (in humans active L1 elements are ~ 6000 nt). Although most of the L1 elements in this analysis are not active, they serve as a genomic “fossil” record of once active elements that can be used to trace L1 evolution across species.

As expected, the number of elements in older families were largely similar amongst all species that shared a common ancestor when the retrotransposon was active: for instance, L1PA7, active prior to the emergence of the last common ancestor of all primates in this study, was found at a relatively constant amount in all genomes (Fig. 4b). On the other hand, human specific elements were found only (barring a few likely mis-annotations) in the human genome. In certain species (gibbon, orangutan and gorilla) instances of retrotransposon families that were active near their divergence from human, were present in much greater copy number than in human (Fig. 4b). Specifically, the number of L1PA4 elements was greater in gibbon then all other apes, while a similar pattern was seen for L1PA3 and orangutan, and L1PA2 and gorilla. These results are consistent with these primates having lineage specific expansion of these elements in a manner distinct from humans. Notably, bonobos had a markedly lower number of L1PA2 elements which may indicate stronger repression of these elements by a species-specific factor; however, the bonobo assembly was one of the older, short-read primate assemblies used in this study, and therefore the lack of L1PA2 elements may simply reflect greater difficulty in resolving these regions in the genome assembly. Note also that the UCSC RepeatMasker track for rheMac10 contains no annotated instances of L1PA5, but this simply reflects the fact that RepeatMasker taxonomy splits the L1PA5 family into L1_RS families that are rhesus-specific compared to the other primates in this study [34]. These L1_RS instances are added to the L1PA5 count in Fig. 4b.

### All apes display evidence of ZNF93 evasion in the 5’UTR of L1PA

In order to examine the selection pressures that might explain species-specific expansion and restriction of L1PA elements, we combined our primate L1PA analysis with the ChIP-seq data of KRAB Zinc Finger Proteins (KZNFs) on the Repeat Browser [26, 35]. KZNFs rapidly evolve in order to directly target retrotransposons and initiate transcriptional silencing of these elements. We previously demonstrated that a 129 bp deletion occurred and fixed in the L1PA3 subfamily (and subsequent lineages of L1PA) in order to evade repression mediated by ZNF93. In order to discover additional cases where a retrotransposon may have deleted a portion of itself to escape KZNF-mediated repression, we searched for L1 sequences with the following characteristics: 1) deletion events proximal to KZNF binding sites, and 2) increasing number of retrotransposon instances with that deletion (demonstrating increased retrotransposon activity). Comparisons of these events across primate species provides evidence for unique, species-specific mechanisms of escape.

**Fig. 5:**
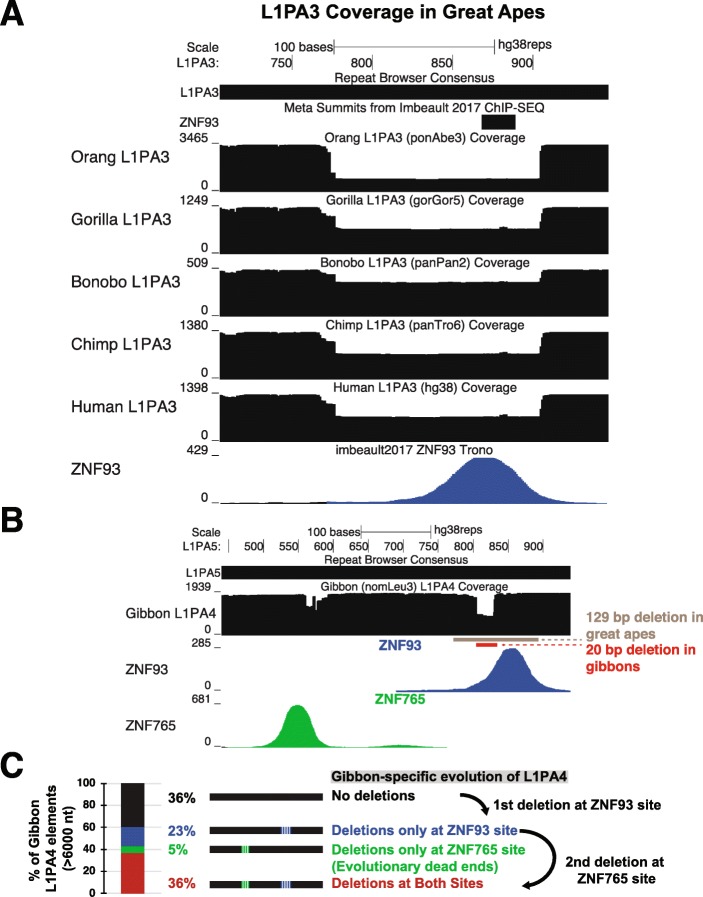
Comparative analysis of L1PA3 elements great apes. **a** Coverage tracks for all full length great ape L1PA3 elements mapped to the human consensus. All great apes exhibit a shared deletion, evidenced by a coverage drop over 129 bp. **b** Coverage map of gibbon L1PA4 elements (mapped to the L1PA5 consensus) demonstrates a different path of ZNF93 evasion (a 20 bp deletion). A second region near the major ZNF765 binding site (green) also demonstrates a coverage drop. **c** Analysis of mutational patterns in gibbon demonstrates that the 20 bp ZNF93-associated deletion likely occurred first in gibbon L1PA4 as most L1PA4s with ZNF765-associated deletions also contain a ZNF93-associated deletion

In order to look for these signatures of L1PA families escaping repression, we used BLAT to align each individual full-length (> 6000 nt) primate L1PA of the same class instance to the human Repeat Browser consensus from the primate genomes under study. We then generated coverage tracks of these full-length elements mapped to the human consensus for each species and each L1PA family. The ZNF93-associated deletion is clearly visible as evidenced by a massive drop in coverage in the 129-bp region in human L1PA3 instances (Fig. 5a). This same drop in coverage is found in all great apes (orangutan, gorilla, bonobo, chimp, and human) confirming that this event occurred in a common ancestor. To explore how more this retroelement lineage may have evolved in more distant primates, we examined gibbon L1PA4 elements on the Repeat Browser and found no evidence of the large 129-bp deletion seen in great apes. Instead, some gibbon L1PA4 elements contain a small 20 bp deletion - at the base of the ZNF93 peak (Fig. 5b). As humans and other great apes do not contain this deletion, we believe that this mutation first occurred in gibbon L1PA4 elements after the human-gibbon divergence. Thus young, gibbon-specific L1PAs may have gained this 20 bp deletion to evade ZNF93 while young great-ape specific elements gained the 129 bp deletion we observed previously [11]. Gibbon L1PA elements with this 20-bp deletion may even hold a selective advantage over the more drastic 129 bp L1PA3 deletions, as elegant work from the Moran lab has recently shown that the 129 bp deletion in human L1PA3 elements alters L1PA splicing in a manner that can generate defective spliced integrated retrotransposed elements (SpIREs) [36]. The smaller deletion found in gibbons may avoid generating these intermediates. Additionally, gibbon L1PA4 elements also experience a smaller coverage drop (typically near the ZNF765 binding site (Fig. 5b). Coverage drops in this area are found predominantly in L1PA4 instances with the ZNF93 binding site already deleted, indicating that this deletion (and the presumed escape from ZNF765 control) occurred after escape from ZNF93 control (Fig. 5c).

### Novel orangutan-specific deletions are visible on the UCSC repeat browser

**Fig. 6:**
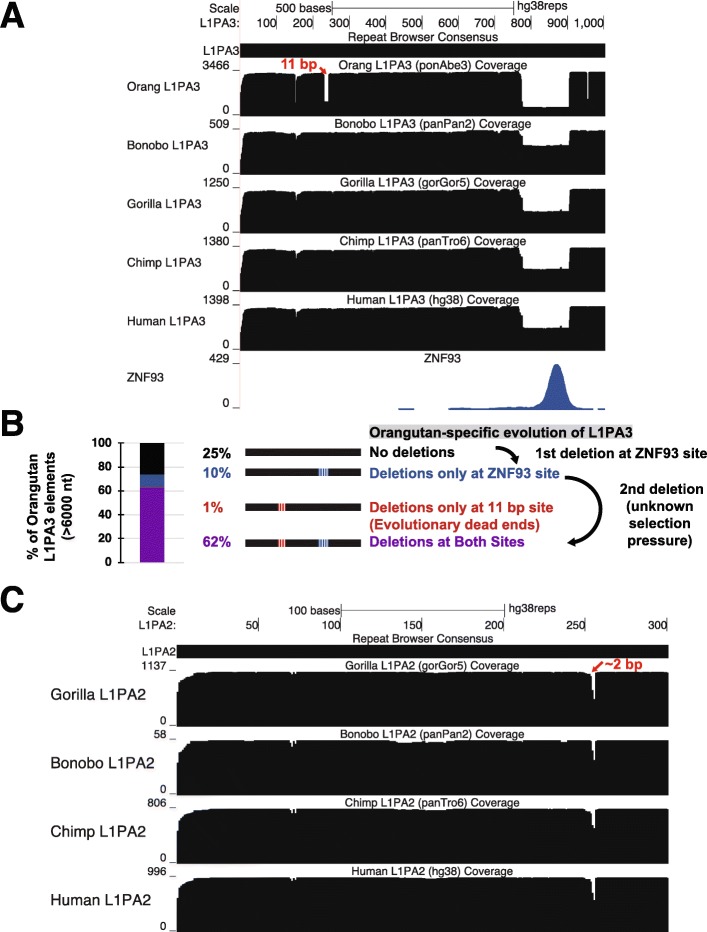
L1PA evolution in great apes. **a** Coverage maps of L1PA3 demonstrate shared deletion of the ZNF93 binding site and an additional 11 bp deletion found only in orangutans. **b** Analysis of the mutational pattern of orangutan elements suggests that the orangutan-specific mutation (red) occurred after ZNF93 evasion (blue) since this mutation is found almost exclusively in elements with the 129-bp deletion already. **c** Coverage map of L1PA2 instances demonstrates no major changes across primates except for small deletions in a region proximal to the orangutan deletion (red)

L1PA3 elements display an increased copy number in the orangutan genome, suggesting that these elements also had a lineage specific expansion, driven by escape from KZNFs or other restriction factors. Aligning of orangutan L1PA3 elements on the Repeat Browser L1PA3 consensus displayed a clear 11 bp deletion ~ 230 bp into the 5′ UTR that is not present in human, chimp or bonobo elements (Fig. 6a). However, analysis of existing KZNF ChIP-seq data, shows no specific factor that clearly correlates with this deletion. We may simply lack ChIP-seq data for the appropriate factor (including the possibility that the KZNF driving these changes evolved specifically within the orangutan lineage) to explain the evolutionary pattern seen in these orangutan-specific elements; alternatively, this mutation might alter some other aspect of L1PA fitness (e.g. splicing). Regardless, L1PA3 elements with this deletion were highly successful in spreading throughout the orangutan genome. Furthermore, L1PA3 instances with deletions in this region also harbor the 129 bp ZNF93-associated deletion, suggesting that this 11 bp deletion occurred after orangutan L1PA3 elements escaped ZNF93 control (Fig. 6b).

### No major deletions are visible in primate L1PA2 elements

Mapping of L1PA2 elements in gorilla, bonobo, chimp and human to the Repeat Browser reveals only minor changes between these relatively young elements. (Fig. 6c) Although gorilla L1PA2 elements have greatly expanded compared to other primates, no significant gorilla-specific deletions are visible in our coverage plots; therefore the spread of gorilla elements may reflect the lack of a control factor that evolved in bonobo, chimpanzees and humans, or may reflect more subtle point mutations as we recently demonstrated for L1PA escape from ZNF649 control [37]. Curiously, all four species show minor coverage drops in the area around nucleotide 250 (Fig. 6c), a region that roughly corresponds to the deletion event observed in orangutan L1PA3 elements. Although the deletion frequencies in primate L1PA2 are relatively low compared to the 11 bp L1PA3 orangutan deletion, this overall behavior is consistent with the model that this region is under adaptive selection - possibly to escape repression from a still unknown KZNF.

## Discussion

**Fig. 7:**
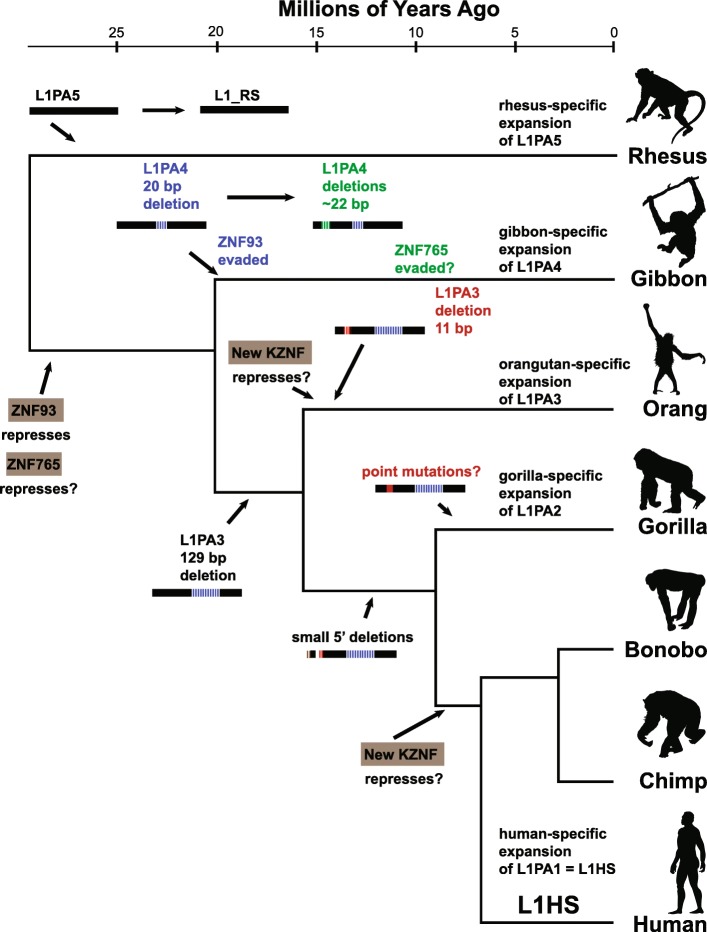
Model for L1PA evolution in different primate species. L1PA5 was active in the ancestor of human and rhesus, and expanded in a rhesus-specific fashion. ZNF93 evolved in the common ancestor of gibbons and humans (ape ancestor) to repress L1PA4 elements. In gibbons L1PA4 escaped with a small 20 bp deletion (blue); a second gibbon-specific deletion event (green) near the ZNF765 binding site led to gibbon-specific expansion of L1PA4. In great apes (human-orangutan ancestor) a 129 bp deletion (blue) in L1PA3 allowed ZNF93 evasion. In orangutans (possibly in response to an orangutan specific KZNF) a new 11 bp deletion occurred and lead to orangutan-specific expansion of L1PA3. In gorillas, continued expansion of L1PA2 is not associated with deletions in the 5’UTR suggesting that this expansion is due either to lack of a chimp/bonobo/human repression factor or point mutations in gorilla L1PA2. A few gorilla, bonobo and human L1PA2 instances experience small deletions (brown and red); the red deletions are in a similar location to the orangutan L1PA3 deletion. Species silhouette images from phylopic.org

The UCSC Repeat Browser provides an interactive and accessible environment to study repeat biology and side-steps the problem of mistakenly mapping reads to an incorrect locus by generating consensus representations of every repeat class, and focusing on how genome-wide datasets interact with repeat sequences independent of their genomic locus. Here we use this consensus-based approach to identify deletion events in repeats across species that suggest a model by which L1PA escape occurs differently across the phylogenetic tree of old world monkeys (Fig. 7). We provide liftOver files so researchers can map their own genome-wide data to the consensus sequences and provide step-by-step instructions at https://repeatbrowser.ucsc.edu/tutorial/.

However, several caveats should be noted about Repeat Browser-based analyses. First, they rely entirely on RepeatMasker classifications (and in turn Dfam) and therefore depend on the quality of the annotations established in these collections. Second, the Repeat Browser often uses its own consensus sequences to display genomic data, with these choices biased by length in order to ensure proper visualization, which can otherwise be problematic in regions where sequence is inserted. However, custom versions of the browser allow users to provide a custom consensus sequence. Indeed, we previously used this approach to create consensuses of L1PA3 subclasses when tracing an evolutionary arms race between ZNF93 and L1PA3 elements [11]. However, the Repeat Browser and other consensus-based approaches risk diluting important, biologically relevant signal driven by a few instances of a repeat type that may affect the cell by virtue of their genomic location instead of their sequence. In these cases, the majority of instances in these families may generate no signal and produce an underwhelming “composite” Repeat Browser signal whereas an individual genomic locus may produce a strong, reproducible, and functionally relevant signal. Therefore, we recommend that Repeat Browser analysis be used in combination with existing genomic approaches for repeat analysis [10, 38–40]. Finally, the existence of the UCSC Repeat Browser as a complete “repeat genome collection” available for download should allow manipulation and utilization of repeat consensus sequences with a large set of existing, standard genomics tools, thereby enhancing the investigation of repeat sequence biology. We expect that the repeat community will make creative use of this tool beyond the workflows suggested here.

## Conclusions

The UCSC Repeat Browser provides a fully interactive environment, analogous to the UCSC Human Genome Browser, to study repeats. We show here that this environment provides an intuitive visualization tool for analysis and hypothesis-generation. For instance, here we use the Repeat Browser to demonstrate that sequence-specific deletions in repeats potentially driven by the activity of cellular repressors occurs independently in different species. The Repeat Browser is currently available at: https://repeatbrowser.ucsc.edu.

## Availability and requirements

**Project name:** The UCSC Repeat Browser.

**Project home page:** https://repeatbrowser.ucsc.edu

**Operating system(s):** Standard Web Browser.

**Programming language:** Python, bash.

**License:** Freely available for academic, nonprofit, and personal use.

**Any restrictions to use by non-academics:** Use of liftOver requires commercial license: http://genome.ucsc.edu/license

**Tutorial:** https://repeatbrowser.ucsc.edu/tutorial/

## Supplementary information

**Additional file 1: Table S1.** Summary of Repeat Browser statistics. This table contains information regarding nomencature changes between the Repeat Browser, RepeatMasker and Dfam as well as several staistics regarding the consensuses and chaining.

## Data Availability

All datasets used are publicly available and are listed in Table 1 and can be downloaded directly on the browser (using the UCSC Table Browser) or on the Repeat Browser website “Data” section (https://repeatbrowser.ucsc.edu/data).
